# Organic-Inorganic Solid-State Hybridization with High-Strength and Anti-Hydrolysis Interface

**DOI:** 10.1038/s41598-018-37052-1

**Published:** 2019-01-24

**Authors:** Tilo H. Yang, C. Robert Kao, Akitsu Shigetou

**Affiliations:** 1 Department of Materials Science and Engineering, National Taiwan University, Taipei, 10617 Taiwan; 2 National Institute for Materials Science (NIMS), Tsukuba, Ibaraki 305-0044 Japan

## Abstract

Organic-inorganic material hybridization at the solid-state level is indispensable for the integration of IoT applications, but still remains a challenging issue. Existing bonding strategies in the field of electronic packaging tend to employ vacuum or ultrahigh temperature; however, these can cause process complications and material deterioration. Here we report an easy-to-tune method to achieve hybrid bonding at the solid-state level and under the ambient atmosphere. Vacuum-ultraviolet (VUV)-induced reorganization with ethanol was used to develop hydroxyl-carrying alkyl chains through coordinatively-bonded carboxylate onto aluminum, whereas numerous hydroxyl-carrying alkyls were created on polyimide. The triggering of dehydration through these hydroxyls by merely heating at 150 °C for a few minutes produced robust organic-inorganic reticulated complexes within the aluminum/polyimide interface. The as-bonded aluminum/polyimide interface possessed an superior fracture energy of (2.40 ± 0.36) × 10^3^ (J/m^2^) compared with aluminum and polyimide matrices themselves, which was mainly attributed to crack deflection due to the nano-grains of inorganic-organic reticulated complexes. The interfacial adhesion was successfully kept after humidity test, which was contributed by those anti-hydrolytic carboxylates. To the best of our knowledge, for the first time organic-inorganic bonding at the solid-state level was achieved using the ethanol-assisted VUV (E-VUV) process, a strategy which should be applicable to a diversity of plastics and metals with native oxides.

## Introduction

**Figure 1:**
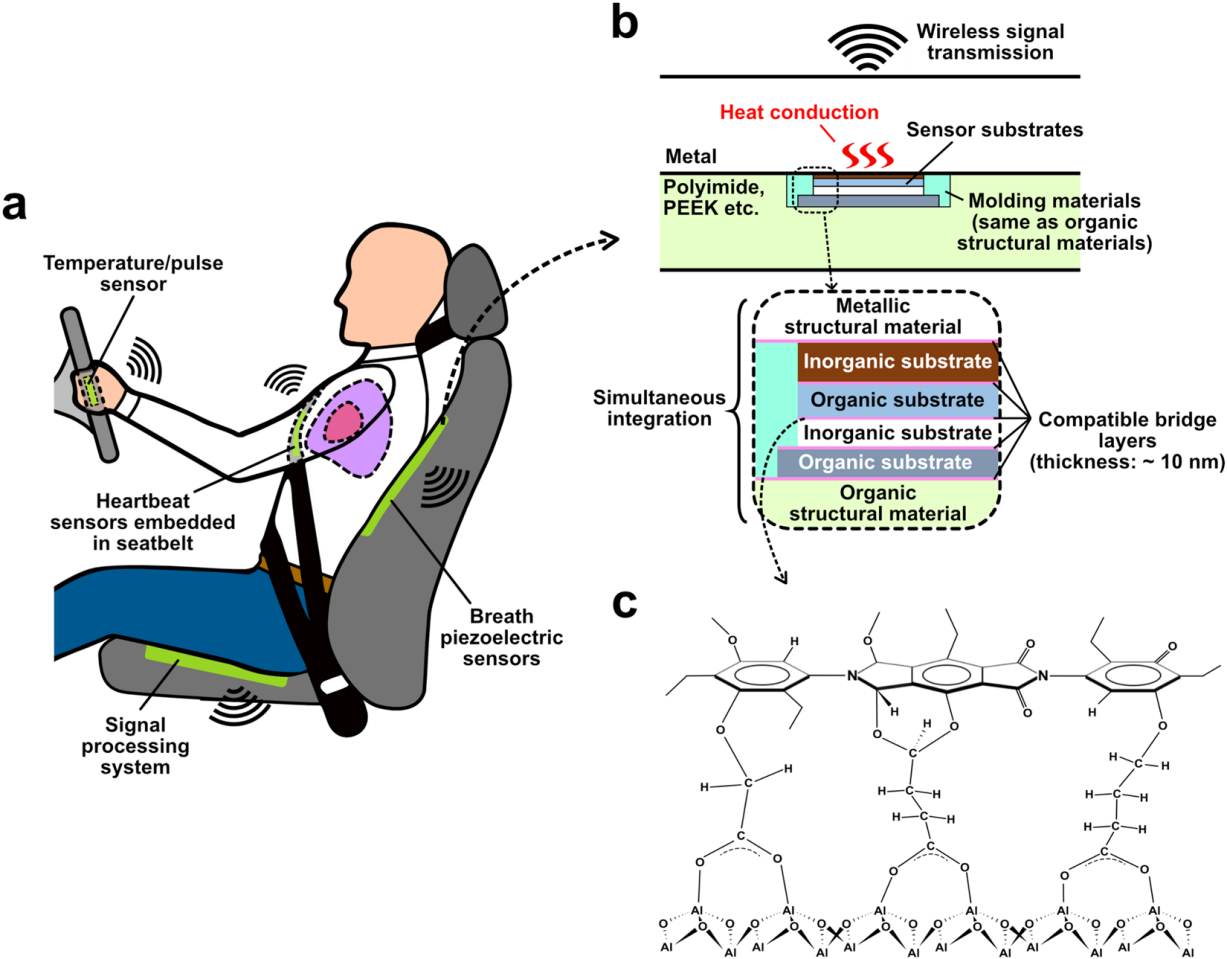
Illustration showing smart and lightweight structural materials via organic-inorganic hybrid integration. (**a**) Illustration demonstrating how an electronics-embedded smart vehicle works for passenger safety. (**b**) Concept of lightweight and smart structural materials where ultrathin electronics packages are involved in structural materials. (**c**) Proposed design of organic/inorganic interfacial architecture with anti-hydrolysis characteristics.

The Internet of Things (IoT) is becoming one of the inevitabilities in the automotive industry, where human safety inside lightweight structural bodies should be monitored continuously via a large number of microelectronic packages, as illustrated in Fig. 1a. For this, seamless signal transmission is necessary between the interior and exterior of lightweight structural materials composed of a combination of metals and organic materials^1–10^. Typical polymers, resins, and some metals are common both to flexible electronic packages and structural materials^1–3,5–11^; therefore, a direct hybrid bonding technology is considered to be highly effective to integrate electronic functions into structural bodies, as illustrated in Fig. 1b. Such electronic packages are conventionally integrated into rigid mechanical modules and assembled with structural materials by mechanical processes such as adhesives and riveting. These techniques are industrially matured; however, their interfaces tend to be a bottleneck for long-term reliability due to the harsh automotive operation environment, such as high temperature, humidity, and exposure to contaminants. Some pioneering studies on laser ablation and friction stir welding have realized organic-inorganic heterogeneous bonding that utilizes high plasticity and diffusivity at temperatures near the melting point^12^, although such high temperatures may be accompanied by considerable thermomechanical damage to the electronic devices. Therefore, to realize lightweight and smart structural materials, the process temperature should be decreased to, for example, the glass-transition temperature (T_g_), which is within the leathery/rubbery plateau region in the viscoelastic behavior of polymers^13–15^. As for low temperature bonding, beam-induced surface activation methods^16,17^ are widely employed for metals and semiconductors; however, these methods cannot be applied to organic and ionically-bonded materials. Most of those techniques also require high vacuum, which results in high process complexity and less compatibility with existing industrial manufacturing facilities. The adsorption of gas molecules is unavoidable in the ambient atmosphere; therefore, the structure of these adsorbate layers must be modified to realize compatible bridging functionality for a diversity of surfaces. In particular, for lightweight structural/wiring metals (e.g., Al, Sn, Cu, etc.) including native oxides, such bridging layers should be created to generate robust ionic or covalent bonds between both of the starting materials. We have previously realized low temperature hybrid bonding in a single process without the need for a vacuum, which was referred to as the vapor-assisted vacuum ultraviolet (V-VUV) method^18,19^. In this method, VUV irradiation with a wavelength of 172 nm was conducted in a humidified nitrogen atmosphere to eliminate surface contaminants, deoxidize the native oxide, and create an ultrathin hydrate layer^18–20^. Such hydrate layers triggered dehydration condensation at temperatures around 100–150 °C upon surface contact, which resulted in strong adhesion via oxygen bridges.

The remaining issue was the long-term reliability of the hybrid materials, especially anti-hydrolysis ability, which is one of the key issues for lightweight structural materials in automotive applications where the materials are exposed to ambient air including certain humidity^21^. There are already matured hydrophobic coating technologies to envelop a hybrid body and make a surface waterproof; however, water absorption of the organic materials from external environment could not be totally avoided in practical operations. Therefore, an effective chemical structure is necessary to prevent interfacial degradation by hydrolysis^22,23^, i.e., the structure of the compatible bridge layer must be designed to keep the reaction with water molecules in equilibrium^22^.

## Bridging Species Selected for Organic-Inorganic Bonding

Given the native oxides of industrial metals, carboxylate is considered as one of the most promising candidates for such a bridging layer due to its reversible reaction with water^24,25^. It has been proven that the coordinatively-bonded carboxylate on an aluminum oxide surface is a thermodynamically stable species even in aqueous environments. The covalent/ionic main molecular chains on the metal surfaces through coordinatively-bonded carboxylates can be dissociated by water molecules via protonation, which may result in crack initiation of the hybrid interface. Nevertheless, for an actual molecular monomer, total delamination of the interface requires simultaneous dissolution of all the bridging carboxylates; therefore, a molecular monomer with more than one bridging carboxylate is able to withstand attack by water (i.e. dynamic competition, see Fig. S1)^25–27^. To achieve such a bridge structure with sufficient tunability of thickness and chemical structure via simple process parameters, we report the design of a low-temperature and atmospheric-pressure hybrid bonding method known as ethanol-assisted VUV (hereafter E-VUV) irradiation (Fig. S2).

In the E-VUV method, multiple exothermic dehydration reactions^28,29^ between complementary species on the polymer and metal are triggered to form the final bridge structure shown in Fig. 1c. The bridging layer is composed of ionic or covalent bonds between both the polymer and metal (oxide) via dehydration reaction upon heating at around T_g_, which results in robust adhesion including multiple carboxylates that resist hydrolysis by water (Fig. S1). VUV light with a wavelength of 172 nm is employed to dissociate ethanol vapor contained in a nitrogen atmosphere to produce H, OH, and CH radical species for the elimination of surface contaminants, partial deoxidization of the native metal oxide, and the formation of hydrophilic functional groups as the trigger for the formation of the bridge layers. The samples require heating to only 150 °C to accelerate dehydration and interdiffusion, and bonding is then completed within a few minutes after the surface contact and subsequent heating. The E-VUV method is conducted under non-vacuum conditions and in a non-toxic environment; therefore, it has potential to be utilized in existing industrial production lines, such as roll-to-roll processes. The evolution of the bridge layer is closely correlated with the amount of exposure to ethanol, Γ (kg·s/m^3^) (see Supplementary Text), which represents the product of the ethanol vapor density (kg/m^3^) and the VUV irradiation time (s). Provided aluminum (Al) and polyimide (PI) as typical examples for lightweight and smart structural materials, it was necessary to clarify the bridge formation behavior.

## Results

### Influence of E-VUV on Aluminum

**Figure 2:**
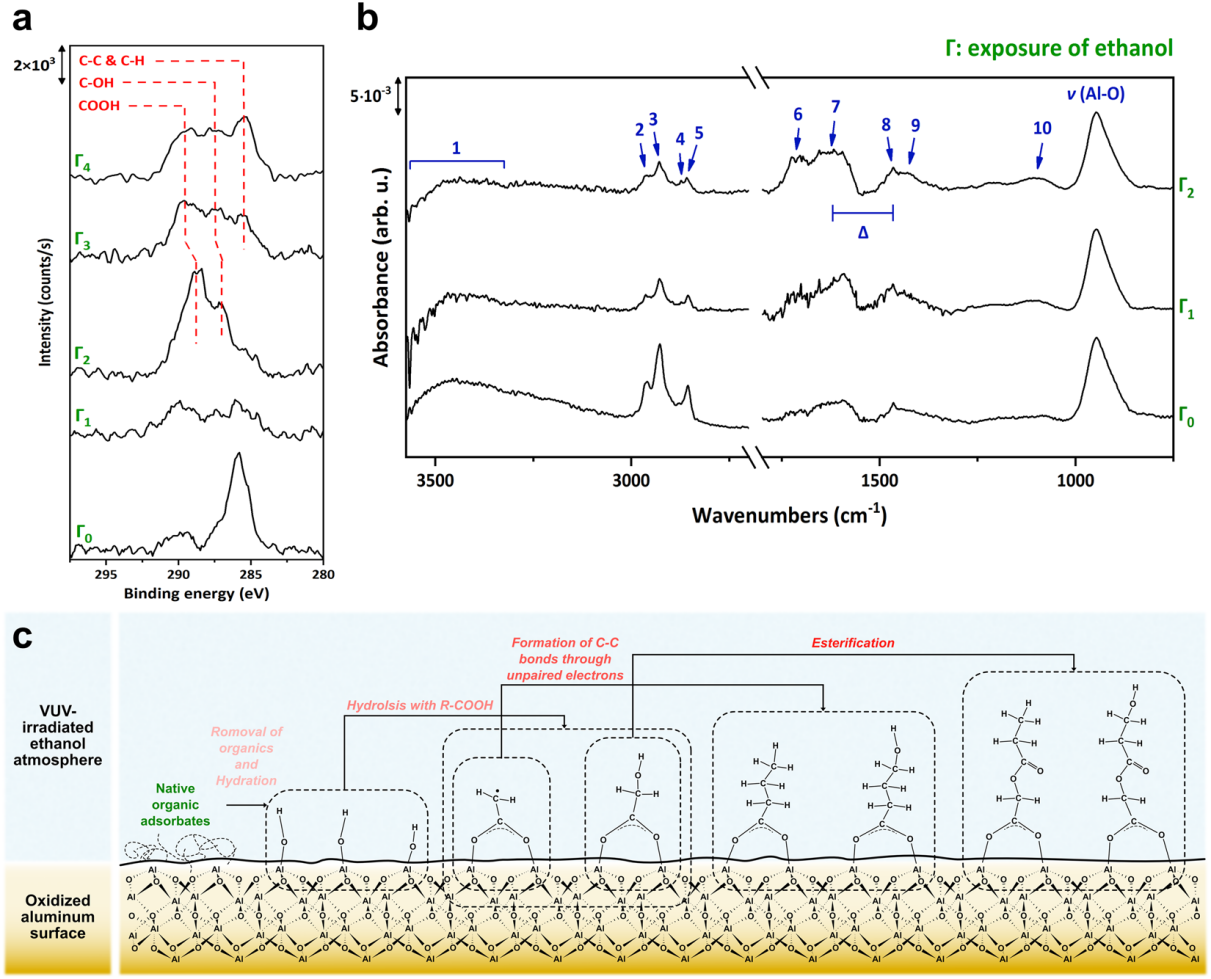
E-VUV-induced reorganization of ethanol present on a metal surface. (**a**) Evolution of XPS C 1s spectra for the aluminum surface subjected to the E-VUV process. The exposure (Γ) of ethanol with a numerical subscript from 0 to 4 represents 0, 218.4, 793.8, 1419.6, and 2087.4 (kg·s/m^3^), respectively. Note that the Γ_2_ spectrum was obtained at a takeoff angle 15° because the intensity at 45° was too low to distinguish chemical components. (**b**) ATR-FTIR spectra for the aluminum surface subjected to the E-VUV process. Γ_0_ to Γ_2_ indicate 0, 436.8, and 2507.4 (kg·s/m^3^), respectively. The symbol Δ denotes the peak separation between symmetric and asymmetric stretching vibrations of carboxyl groups, and is around 151 cm^−1^ in these spectra. (**c**) Illustration showing how the bridging layer forms on the aluminum surface with the E-VUV process.

We first analyzed the effects of E-VUV on polycrystalline Al films, which were fabricated on a silicon wafer by electron-beam deposition. The difference in the chemical bonding status after VUV irradiation at various exposures was evaluated using X-ray photoelectron spectroscopy (XPS) and attenuated total reflection Fourier transform infrared spectroscopy (ATR-FTIR). Figure 2a,b show the evolution of the XPS and ATR-FTIR spectra (see peak assignments in Table 1). Organic contaminants were almost eliminated when the E-VUV exposure was low (see spectrum Γ_1_ in Fig. 2a,b), which is attributed mainly to the reaction of hydrogen and oxygen radicals with the organic contaminants to convert them into gaseous CO_2_ and H_2_O^30^. After cleaning, the alumina surfaces were gradually hydrated by the formation of hydroxyl groups on the Al sites (spectrum Γ_1_ in Fig. 2b; see also Fig. S3). With increased exposure to E-VUV, the VUV-induced molecules with carboxylic groups were deprotonated through these hydroxyl groups on the alumina surface to form coordinatively-bonded carboxylate species (see spectrum Γ_2_ in Fig. 2a,b). The coordination type of these carboxylate ligands was clarified by measurement of the peak separation between symmetric and asymmetric stretching vibrations of carboxylate in the ATR-FTIR spectra. The peak separation (∆) was measured to be around 151 cm^−1^, which indicates that the carboxylates were coordinated to two aluminum cations in the surface alumina in the conformation of a bridging bidentate ligand^31–34^. Hydroxyls were formed at the chain terminals of those carboxylates (Figs 2a and S3). As E-VUV exposure increased, acyclic alkyl and ester groups were then produced (spectrum Γ_4_ in Fig. 2a, spectrum Γ_2_ in Fig. 2b, and Fig. S3). This was probably due to the hydroxyl groups on the carboxylates that underwent esterification through the gaseous molecules carrying carboxyls, which resulted in alkyl grafting. Another subsidiary reason for the increase in the C-C signal was that VUV irradiation produced many unsaturated carbon atoms on the surface carboxylates by cleaving C-H bonds, so that the unsaturated carbon atoms attracted unsaturated gaseous alkyl groups through unpaired electrons and were then transformed into saturated C-C bonds. From these results, the E-VUV process was demonstrated as effective for the generation of acyclic species carrying hydroxyl or alkyl end groups onto the alumina surface through bridging carboxylate (Fig. 2c).

**Table 1:** Peak positions and corresponding assignments of the numbers marked in Fig. 2b.

No.	Wavelength (cm^−1^)	Assignment	No.	Wavelength (cm^−1^)	Assignment
1	3338–3504	*ν*(OH)	6	1703	*ν*(C=O) of COOH
2	2954	*ν*_*as*_(CH_3_)	7	1616	*ν*_*as*_(COO^−^)
3	2929	*ν*_*as*_(CH_2_)	8	1465	*δ*(CH_2_)
4	2870	*ν*_*s*_(CH_3_)	9	1430	*ν*_*s*_(COO^−^)
5	2856	*ν*_*s*_(CH_2_)	10	1108	*ν*(C−C−O) of -CO_2_R ester

### Influence of E-VUV on Polyimide

**Figure 3:**
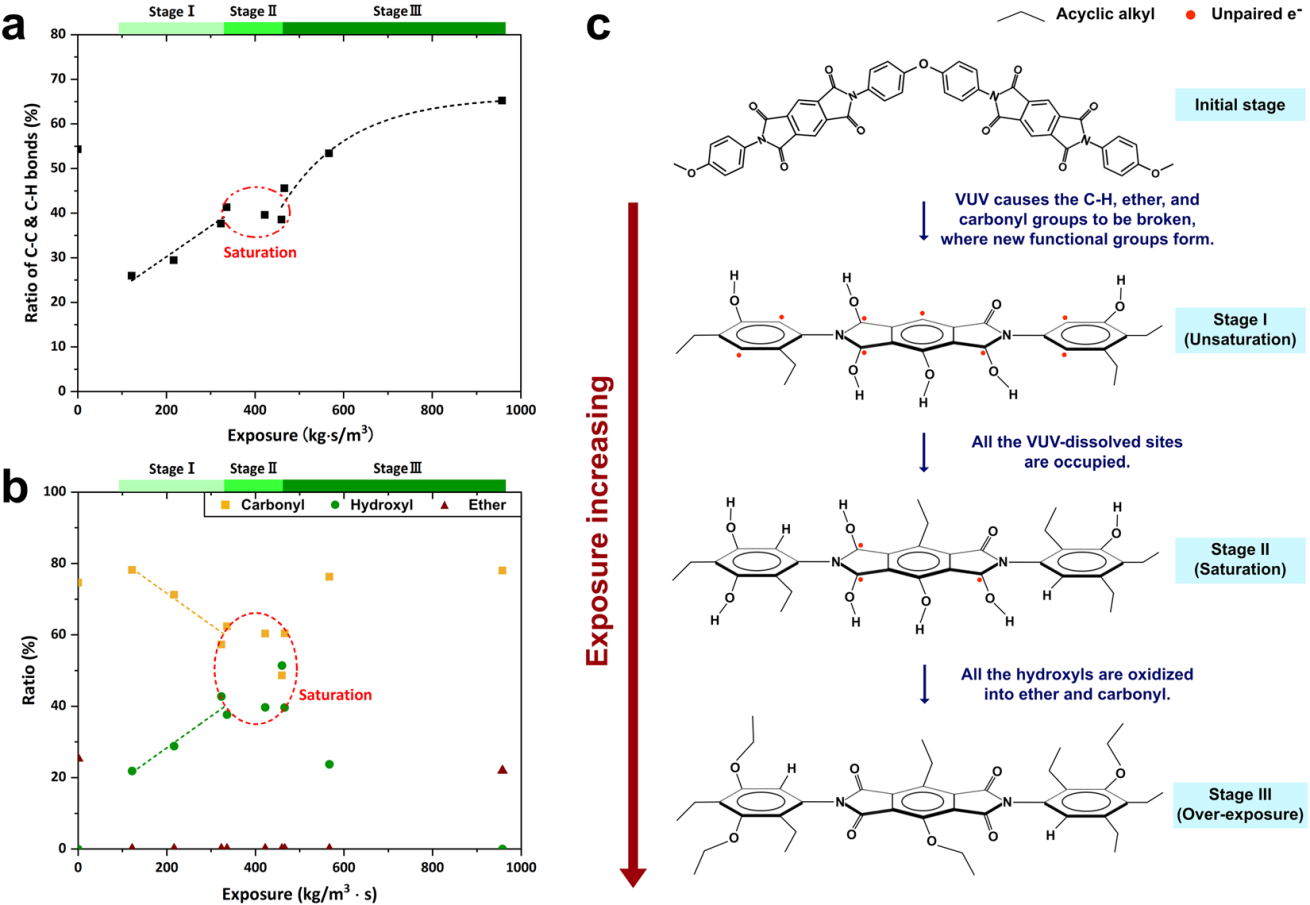
E-VUV-induced reorganization of ethanol present on a polymer surface. (**a**) Change in the ratio of carbon-carbon and carbon-hydrogen bonds on PI with increasing exposure to E-VUV. (**b**) Change in the relative ratio of carbon-oxygen functional groups among hydroxyl, carbonyl and ether groups. (**c**) Proposed mechanism for E-VUV-induced grafting on the PI surface.

Similar analyses were conducted on PI films (PMDA-ODA, DuPont Kapton). Figure 3a,b reveal that there were three stages in the evolution of the surface chemical structures (for which the original XPS spectra are shown in Figs S4 to S6). At the initial stage of reaction, the ratios of hydroxyl and alkyl groups increased in accordance with the amount of exposure, which implied that the hydroxyl and alkyl species were gradually grafted onto the sites of free radicals, i.e. dangling bonds, which were generated by VUV irradiation (stage I in Fig. 3c). The linearity of grafting is comprehensible based on the kinetic theory of gases^35^:

1 $${N}^{\ast }=({N}_{A}P/\sqrt{2\pi RMT})\cdot t,$$

where N^*^ represents the number of gas molecules that collide with a unit area of a surface, *N*_*A*_ is the Avogadro constant, *P* is the gas pressure, *R* is the gas constant, *M* is the molecular weight, *T* is the temperature, and *t* is the exposure time^35^. Assuming that *P* and *t* are the only two variables in the E-VUV process, the grafting number of hydroxyl and alkyl groups was proportional to the product of *P* and *t* (i.e. the exposure of ethanol), which implies that the formation of chemical bonds by sharing unpaired electrons was dominant for grafting at this stage. While all the sites of the free radicals on the PI surface were occupied by hydroxyl and alkyl groups, grafting showed metastable behavior (stage II in Fig. 3c), which was evident from the constant ratio of every chemical component within 320–470 (kg·s/m^3^). The ratio of hydroxyls dropped sharply with increased exposure to E-VUV, whereas that of carbonyls and ether increased significantly (Fig. 3b). The ratio of alkyl chains increased following a logarithmic law and eventually became saturated (Fig. 3a; see also Fig. S7). All the hydroxyl groups on the PI chains were still reactive at this stage, so that the hydroxyls at imide rings could be further oxidized to carbonyl groups, and other hydroxyls could react with hydroxyl-carrying gaseous molecules to be transformed into ether linkages with alkyl chains (stage III in Fig. 3c). The presence of hydroxyls is thus a key factor to induce dehydration reaction^20,36^; therefore, E-VUV exposure at stage II is considered the optimum condition for hybrid bonding.

### Interfacial Microstructures and Chemical Configuration

**Figure 4:**
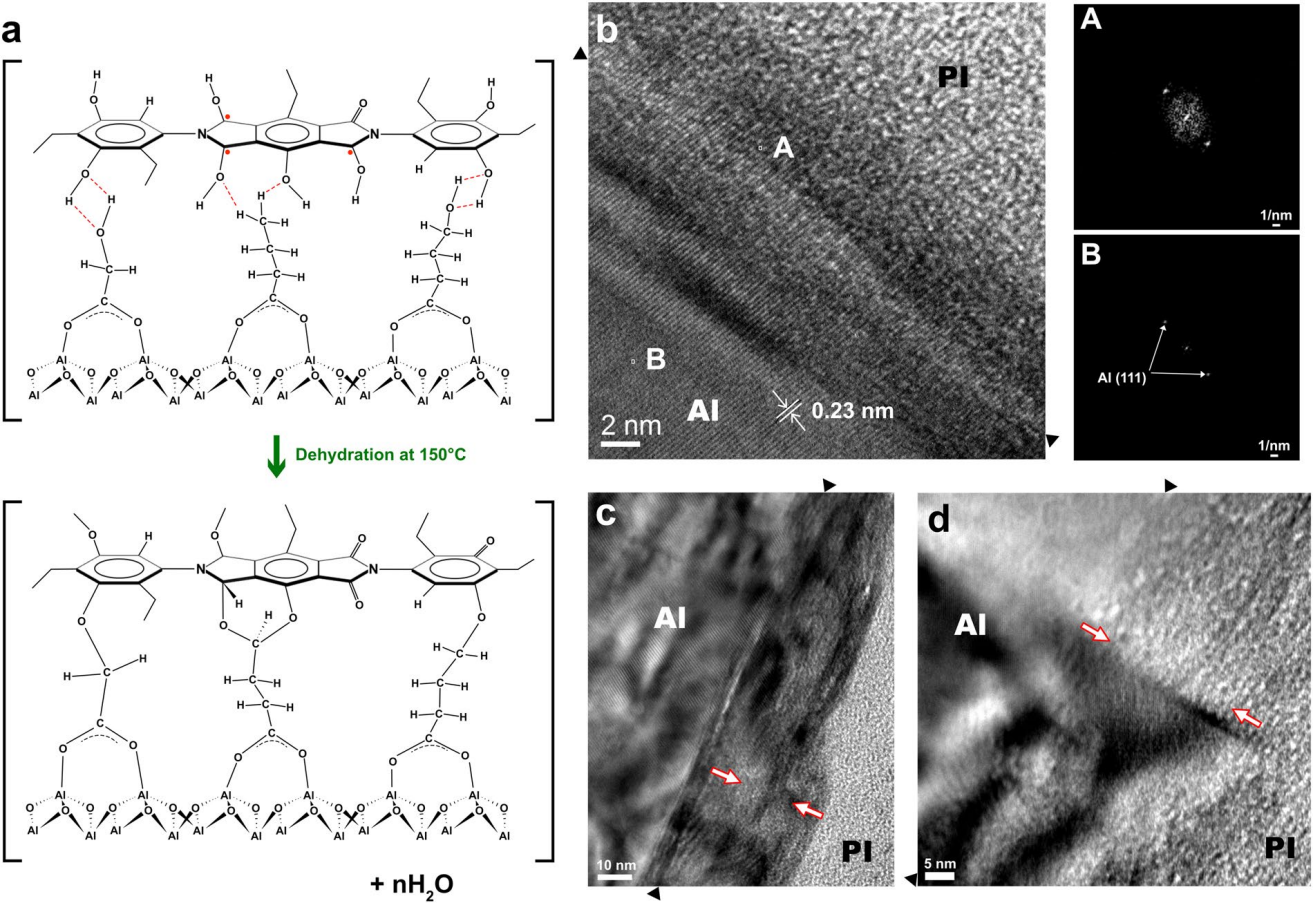
Formation and microstructure of heterogeneous Al/PI interface. (**a**) Proposed mechanism for the formation of a heterogeneous Al/PI interface through dehydration reaction at 150 °C. (**b**) High-resolution TEM image of the as-bonded Al/PI interface after bonding at 150 °C for 10 min. Diffraction patterns of the Al matrix and the interface reveal that the interface, where some new fine crystals are formed, grew after crystal orientation of the Al matrix. (**c**) and (**d**) show the interfacial microstructure after storage at room temperature for 3 and 6 months, respectively. The symbols ▲ and  point out the initial interface where Al and PI surfaces were brought into contact and the Al/PI interfacial region, respectively.

Based on the optimized conditions (Fig. S2c), hybrid bonding was successfully achieved between Al and PI surfaces. Figure 4a shows a schematic representation for the formation of the Al/PI interface via the ultrathin bridge layer created during the E-VUV process. It should be noted that water molecules generated during dehydration are expected to escape from the interface due to their considerably small molecular volume compared with free volume of polymers. Figure 4b shows a transmission electron microscopy (TEM) image of the Al/PI interface, which was obtained after heating at 150 °C for 10 min (the heating time was equipment-dependent). In Fig. 4b, the metal lattice fringes (d_(111)_ spacing = 0.23 nm) extended continuously from the bulk Al matrix side to the PI side via the interface, which indicates that hybrid bonding was achieved without distortion of the matrix of starting materials (Al) due to the considerably thin interfacial bridge layer with a thickness of less than 3 nm (see also the diffraction patterns in Fig. 4b). Figure 4c,d show TEM images of the bond interface after isothermal aging at room temperature for 3 and 6 months, respectively. The thickness of the interfacial region was increased (Fig. 4c) with time and its growth became saturated at around 35 nm (Fig. 4d; see also Fig. S8) with the formation of a composition-gradient layer through successive interdiffusion from Al to PI via the ultrathin bridge layer.

**Figure 5:**
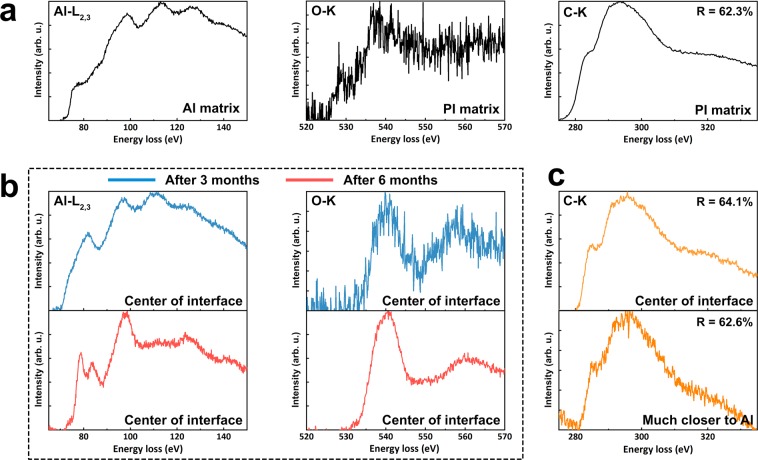
Evolution of chemical configuration of the Al/PI interface with storage at room temperature. (**a**) EELS Al L_2,3_-edge from the Al matrix, and C- and O-edges from the PI matrix after hybrid bonding. (**b**) EELS Al L_2,3_- and O-edges from the center of the Al/PI interface after storage at room temperature for 3 and 6 months. (**c**) EELS C-edge from the center of the interfacial region (i.e. a position ca. 10 nm away from the native alumina) after storage at room temperature for 6 months (upper), and that obtained at a position ca. 5 nm away from the native alumina (lower). The ratio (R) marked at the upper right corner in each figure represents the intensity ratio I_σ*_/(I_σ*_ + I_π*_), where I_σ*_ and I_π*_ are the signal intensity of σ* and π* bonds in each C-K spectrum, respectively.

A series of electron energy loss spectroscopy (EELS) analyses were conducted to determine the chemical configurations of this interfacial region. Figure 5 shows the core-shell spectra of aluminum (Al-L_2,3_), oxygen (O-K), and carbon (C-K) taken from the very interface and deep inside the matrices, respectively. The Al-L_2,3_ spectra indicate that interfacial Al remained mainly oxidized after 3 months, even though considerable unoxidized Al atoms were evident (Fig. 5b). Neutral Al atoms were barely observed after 6 months, and two clear peaks corresponding to Al-O bonds appeared at ca. 80 eV^37–39^, which also supports the results from TEM observations. A corresponding sharp peak of metal oxide at 540 eV was observed in the O-K spectra (Fig. 5b)^37^. It is considered that the peak at ca. 558 eV was due to a decrease in the symmetry of the oxygen sites of alumina crystals; such as a decrease in long-range ordering or an increase in the variance of neighboring Al-O bond lengths. The latter factor is supported by the appearance of a peak at ca. 537 eV in Fig. 5b, which was attributed to the σ* hybrid state for carbon-oxygen single bonds^39,40^, and implied the interfacial alumina crystals and surrounding molecules of PI formed an organic-inorganic composite though Al-O-C bonds. In addition, peaks of hydrated Al^41^ were barely observed in the O-K spectra, which supports the fact that the water molecules forming from dehydration reaction might escape from the Al/PI interface. The interfacial region exhibited a much higher concentration of the σ* hybrid state than the PI matrix (Fig. 5c), which implies that the interfacial carbons were organized mainly with a network-like texture. In contrast, the C-K spectrum obtained at a point much closer to the Al region showed a greater concentration of the π* state (Fig. 5c). The increase in the concentration of the π* state may result from the sp^2^ hybrid state of the carboxylate on the native alumina layer, which is consistent with the results shown in Fig. 2c.

### Evaluation of Interfacial Adhesive Strength and anti-hydrolysis characteristic

**Figure 6:**
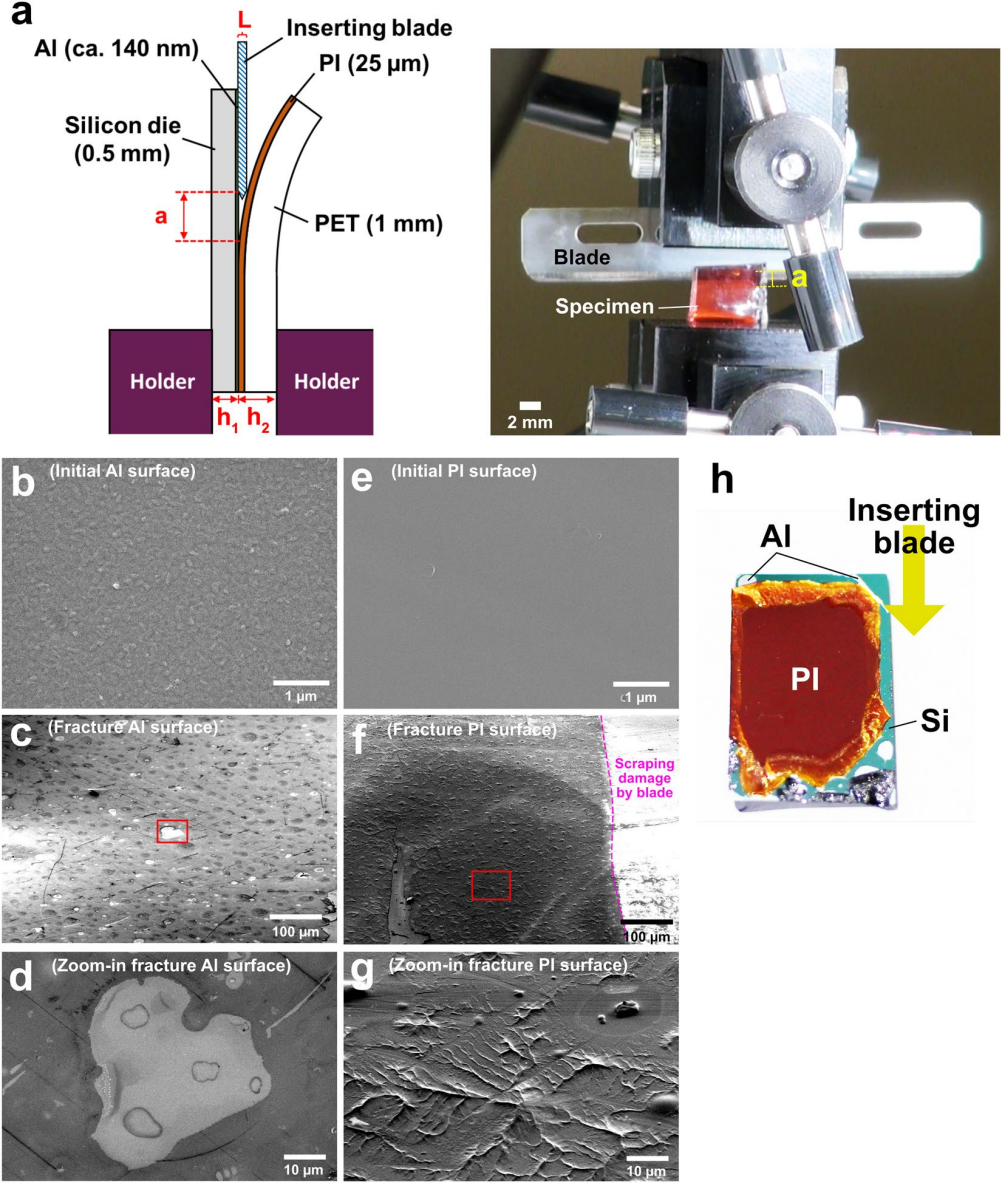
Evaluation of fracture energy and anti-hydrolysis property of the Al/PI interface. (**a**) Illustration showing how the interfacial fracture energy was quantitatively measured via an asymmetric double cantilever beam (ADCB) test. The right photograph showing how the ADCB test was actually conducted. (**b**) to (**d**) Initial aluminum, fracture aluminum surface, and zoom-in image of  in (**c**). (**e**) to (**g**) Initial polyimide, fracture polyimide surface, and zoom-in image of  in (**f**). All the fracture surfaces were taken from crack tip ahead of the blade. To enhance topographical contrasts, (**c**), (**f**) and (**g**) were taken at a tilt angle 30°. (**h**) Photograph showing the ADCB-tested specimen after the temperature humidity storage (85 °C/85RH%) for 1000 h. The blade was inserted from the upper edge of the specimen down into the interface.

The Al/PI interfacial adhesive strength was evaluated in terms of the fracture energy required to produce an increment of new surface as a razor blade was advanced along the Al/PI interface during an asymmetric double cantilever beam (ADCB, for which experimental details are depicted in Methods) measurement, as illustrated in Fig. 6a. The fracture energies measured by such the blade testing were considered to approximate to the sum of surface energy of two solid beams. Comparison of the fracture energy measured by the ADCB test with those reported in previous studies are summarized in Table 2. The ADCB results show that the as-bonded Al/PI interface possessed an extremely high fracture energy of (2.40 ± 0.36) × 10^3^ (J/m^2^), which implies that the heterogeneous interface achieved by the E-VUV process had better toughness to resist fracture than bulk Al and PI matrices themselves. Some common wafer bonding techniques like plasma activation are also listed in Table 2. Although the differences in measuring methods and elasticity/plasticity of materials led to large variations in fracture energy in the previous studies, the E-VUV method is considered to be sufficiently capable of creating ultrahigh fracture energy in flexible electronics packaging. On the basis of the TEM and EELS analyses, it is believed that Al was the dominant species participating interdiffusion from Al to PI via the ultrathin bridge layer. A considerable amount of nanograins of an organic-inorganic network-like complex was then formed within the interface through coordinated-bonded carboxylates on the native alumina by the E-VUV process, which is also implied by the new diffraction spots in the Al/PI interface compared with that of the Al matrix (see Fig. 4b). The nanoscale grains of reticulated complexes resulted in organic/inorganic interfaces that were tough due to crack deflection^42–45^ by the strong complex grains during crack propagation. Figure 6b–g compare the initial and fracture Al and PI surfaces. The mode of adhesion fracture was observed, and the fracture Al and PI surfaces became rougher compared with the initial surfaces, which supports the interface toughening mechanism.

**Table 2:** Summary of interfacial fracture energy from literature and this study.

Interface type	Fracture energy (J/m^2^)	Measuring technique	Sample description and literature source
Al/PI	(2.40 ± 0.36) × 10^3^	ADCB	E-VUV-assisted bonding at 150 °C for 10 min; no post-annealing; this study.
Al/Al	2.40	Theoretical calculation	Thermodynamic work of cohesion; DFT calculation; (111) plane^59^.
	11.92 ± 1.86	DCB	Direct bonding at 200 °C for 10 min after Ar-FAB treatment; no post-annealing; this study.
Al_2_O_3_/Al_2_O_3_	7.42–7.96	Theoretical calculation	Thermodynamic work of cohesion; ab initio calculation; unrelaxed α-Al_2_O_3_ basal plane^60^.
	4.14 ± 0.24	DCB	ALD growth on Si wafer; room-temperature direct bonding after plasma activation, and subsequently annealed at 200 °C for 2 h^44,61^.
PI/PI	7.44 × 10^−2^	Theoretical calculation	Thermodynamic work of cohesion; Dupont Kapton (PMDA-ODA)^62^.
	(3.31 ± 0.64) × 10^−3^	DCB	Direct bonding at 150 °C for 10 min after water-vapor-assisted VUV treatment; no post-annealing; this study.
Silicon wafer bonding	4.26	Theoretical calculation	Thermodynamic work of cohesion; (100) plane^63^.
	~4.50	DCB	Direct bonding via sequential plasma activation; room-temperature storage for 24 h^44,64^.
	2.20–2.60	DCB	Room-temperature direct bonding after standard RCA cleaning; hydrophilic surface; post-annealing at 150 °C for 20 h^44,65^.
	3.80–4.20	DCB	Room-temperature direct bonding after TMOS modification; post-annealing at 150 °C for 20 h^44,65^.
	4.00–4.60	DCB	Direct bonding at 1400 °C for 10 min after sprayed with deionized water^44,58^.
	2.50–3.00	DCB	Direct bonding at 1200 °C for 10 min after soaked in NH_4_OH^44,58^.

Thermodynamic work of cohesion^66^ (*W*_*c*_) was calculated based on *W*_*c*_ = 2*γ*, where *γ* is the surface energy of solids.

Figure 6h demonstrates the Al-PI specimen after the temperature humidity test (85 °C/85RH%, no bias). The Al/PI interface survived the reliability test, which indicates that the proposed carboxylate-containing interfacial structures effectively kept interfacial adhesion. As also shown in Fig. 6h, since the Al/PI interface became much stronger after the reliability test, the crack was deviated into the PI matrix instead of propagation along the Al/PI interface. The microstructural evolution of organic-inorganic interface with the temperature humidity test is of interest to verify strengthening mechanisms.

Compared with Ar-FAB and VUV process, which has been discussed in our previous studies^7,20^, the E-VUV method was conducted at comparably low temperatures and a non-vacuum atmosphere. Moreover, the E-VUV process was able to create robust heterogeneous interfaces within few minutes without post-annealing. The E-VUV process first removed almost all of native organic contaminants on Al surface at the low-exposure stage so that the coordinatively-bonded carboxylates could be formed and firmly adhered on Al oxide surface, and then produced a great number of surface hydroxyls that were transformed into strong covalent bonds during hybrid bonding, both of which enhanced bondability significantly.

## Outlook

The E-VUV process provides access to the development of organic-inorganic solid-state integration. Given that native oxides are present on industrial metals and that the VUV photon energy is sufficient to fragment surface molecules in most engineering plastics, the E-VUV process should have broad applications in cross-scale material integration, such as in nanophotonics^46,47^, perovskite solar cells^48^, and smart structural materials^5,7^. The bonding temperature may be further reduced based on the fact that hydroxyls on hydrophilic surfaces are capable of undertaking dehydration at low temperatures if they are close enough and are hydrogen-bonded^36^. The organic-inorganic network-like complex contributes to a robust and tough hybrid interface, whereas the coordinatively-bonded carboxylates on the same monomer maintain long-term interfacial reliability against hydrolysis by water. Further design of interfacial bridging architectures is expected to become a key for the realization of strong and highly-reliable heterogeneous interfaces.

## Methods

### Materials

For aluminum/polyimide hybrid bonding, a polycrystalline aluminum thin film with a thickness of 140 nm was pre-deposited onto a silicon wafer using an electron-beam evaporation system (UEP-3000-2C, Ulvac Inc.) and prepared to dimensions of 10 × 10 mm^2^. A polyimide (PI) thin film (PMDA-ODA, DuPont Kapton) with a thickness of 25 µm was prepared to dimensions of 15 × 15 mm^2^. Prior to use, all the aluminum samples were ultrasonically cleaned for 3 min each in acetone, ethanol, and deionized water. All the PI films were ultrasonically cleaned for 3 min each in ethanol and deionized water. In order to make a reference Al-Al specimen for comparison, we used a commercial aluminum foil (Alfa Aesar Inc., 99.99% purity) with thickness 0.1 mm. The cleaning procedure was as mentioned above.

### E-VUV Surface Modification and Hybrid Bonding

To conduct *in situ* evaluation of the surface chemical condition after ethanol-assisted VUV (E-VUV) treatment, an in-house-built apparatus was developed (Fig. S2a), which comprised three chambers for VUV irradiation, X-ray photoelectron spectroscopy (XPS) analysis, and hybrid bonding, respectively. In this apparatus, the VUV and XPS chambers were mutually connected with the vacuum condition. For that reason, the surface analysis was conducted *in-situ* using XPS.

The samples were placed into the VUV chamber with a background vacuum of 10^−4^ Pa. A nitrogen atmosphere containing ethanol (99.5 vol%, Wako Chemicals, Ltd.) was introduced into the VUV chamber using an ultrasonic atomizer until the gas pressure reached 9 × 10^4^ Pa (ca. 0.9 atm). During the E-VUV treatment, the vapor humidity in the VUV chamber was recorded and temperature was kept around 24 °C. A VUV source with a wavelength of 172 nm (UER20H-172VA, Ushio Inc.) was used to provide VUV irradiation and its incident power per area was kept at 5 mW/cm^2^. The sample surface was ca. 70 mm away from the VUV source and the irradiated region was within a circle with a radius of ca. 10 mm. After the E-VUV irradiation treatment reached pre-determined exposure (for which the value was determined on the basis of XPS and attenuated total reflection Fourier transform infrared spectroscopy (ATR-FTIR) results; see also Fig. S2c), the E-VUV-treated specimens were transferred into the bonding chamber using robotic arms. The lower stage in the bonding chamber was installed with a heater. After mating samples were brought into contact with each other, a load of 400 N, of which the pressure equaled 4 MPa according to the specimen dimensions, was given to the hybrid couple for sufficient surface contact, followed by isothermal bonding at 150 °C for 10 min.

### Angle-Resolved X-ray Photoelectron Spectroscopy (ARXPS)

The samples subjected to the E-VUV treatment were sequentially transferred into the XPS chamber to evaluate the effects of the surface treatment. XPS analysis was conducted *in situ* using non-destructive detection with a Mg Kα (1256 eV) source employed at 27 mA and 15 kV. The operating power was 400 W. Three of the takeoff angles (θ) were 15°, 30°, and 45°, where θ = 90° agrees with the normal to the surface. Analysis of XPS spectra was performed using PHI Multipak software. Peak deconvolution was conducted by fitting Gaussian-Lorentzian mixed functions.

### Attenuated Total Reflectance Fourier Transform Infrared Spectroscopy (ATR-FTIR)

ATR-FTIR measurements were performed using a Thermo Scientific Nicolet 4700 spectrometer in total external reflectance mode. An infrared beam was transmitted through KBr windows onto the sample, and reflected at an incident angle of 80° to a mercury-cadmium-tellurium (MCT) detector cooled with liquid nitrogen. The spectrometer was kept purged with nitrogen gas during the measurement. Spectra were collected at a resolution of 2 cm^−1^ and with the coaddition of 128 single scans.

### Observation of Al/PI Interface

Transmission electron microscopy (TEM) and electron energy loss spectroscopy (EELS) were conducted for observation of the interfacial microstructures/chemical configurations. TEM samples were prepared using a focus ion beam (FIB) system (JIB-4000, JEOL) with a Ga liquid-crystal ion source. TEM and EELS investigations were conducted using a FEI Tecnai G2 F30 installed with parallel EELS (PEELS) operated at 300 kV. The energy resolution was measured to have a full width at half maximum (FWHM) of 1.3 eV on the zero-loss peak. To record the energy-loss near-edge structure (ELNES), a dispersion of 0.1 eV/channel, an entrance aperture diameter of 2 mm, and a cumulative time of 10.24 s per acquisition were employed.

### Assessment of Interfacial Adhesion

An asymmetric double cantilever beam (ADCB) test was conducted to estimate fracture toughness of the Al/PI interface, as portrayed in Fig. 6a. An universal testing machine (LTS-B Series, MinebeaMitsumi Inc.), where an inserting blade and samples under test were installed on the upper and lower stages, respectively, was employed in compression mode to conduct ADCB tests. All of the energy released during fracture was assumed to be consumed only locally near the crack tip in an interfacial zone which was small compared with the thickness of two beams. Such an assumption allowed the fracture toughness, *G*_*c*_ (J/m^2^), to be equal to the energy release rate, *G*, of the beam (substrate) obtained by a simple relationship derived from beam theory^49–53^.

2 $${G}_{c}=\frac{3}{8}\frac{{L}^{2}}{{a}^{4}}\frac{{E}_{1}{h}_{1}^{3}{E}_{2}{h}_{2}^{3}}{{E}_{1}{h}_{1}^{3}+{E}_{2}{h}_{2}^{3}}$$

where *L* represents the blade thickness; *a* is the crack length; *E* is Young’s modulus of the beam materials, and *h* is the thickness of the Al and PI beams; the subscripts 1 and 2 represent the aluminum and polyimide beams.

Equation (2) has been experimentally and numerically proven reasonably valid as the beam geometry was kept 0.5 ≤ *h*_2_*/h*_1_ ≤ 2, where *h*_1_ is the thickness of the stiffer material compared with the other^51,52,54^. There are two precautions we need to obey. The first one is that mechanical asymmetry of the beams (*E*_1_*/E*_2_ ≫ 1) would lead to overestimation of *G*_*c*_ and *G* due to a large non-zero phase angle at the crack tip which results in the crack deviating into the ductile side from the interface^50–52,54–56^. To avoid this, the Al beam, which was the stiffer material in this study, should be made thinner than the PI beam. Given the fact that Young’s modulus of PET is almost the same as PI, we adjusted thickness ratio by adding a transparent PET tape on the PI film. The overall specimen geometry used for ADCB test was marked in Fig. 6a. The *h*_2_*/(h*_1_ + *h*_2_) was measured to be 0.672, which was proved to effectively suppress the effect of shear component *K*_*II*_ for polymer/inorganic interfaces^51,52,54^. Because of close *E* values of PI and the polymers used in those studies^52^, this thickness ratio was expected to be able to direct crack propagation right along the Al/PI interface in this work.

The second precaution is that equation (2) is only appropriate for the interfacial fracture estimation of weak interface with long cracks. For stronger interface with shorter cracks, this equation would lead to overestimation of *G*_*c*_ and *G*. On the basis of correction done by Kanninen^53^, the core assumption was that correction factors required being introduced in the finite elasticity of the material ahead of the crack tip for the case of short crack length. According to this model, *G*_*c*_ was corrected to be^51,52^

3 $${G}_{c}=(\frac{3{E}_{1}{E}_{2}{h}_{1}^{3}{h}_{2}^{3}{L}^{2}}{8{a}^{4}})[\frac{{C}_{2}^{2}{E}_{1}{h}_{1}^{3}+{C}_{1}^{2}{E}_{2}{h}_{2}^{3}}{{({C}_{2}^{3}{E}_{1}{h}_{1}^{3}+{C}_{1}^{3}{E}_{2}{h}_{2}^{3})}^{2}}]$$

where

4 $${C}_{1}=(1+0.64\frac{{h}_{1}}{a})$$

5 $${C}_{2}=(1+0.64\frac{{h}_{2}}{a})$$

Thus, equation (3) provides a more accurate approximation for *G*_*c*_. This approximation stays valid if the crack length to ratio of beam thickness is large (i.e. *h*_*i*_*/a* < 1) and the length of non-fractured interface ahead of the crack tip is large compared with the beam thickness.

In this study, a blade (Captain blade, Kai Ltd.) with thickness of 0.25 mm was inserted at the Al/PI interface and was pushed into it at a low and constant velocity of 25.0 μm/s so that the measured energy release rate could be assumed to equal the critical energy release rate (*G*_*c*_) at a zero velocity. The crack length ahead of the blade was recorded by a video camera observing through the translucent PI side. At least ten screen captures were taken per specimen and crack length measurement was conducted at three different locations in each captured figure to obtain a mean crack length for the estimation of fracture energy. The deviation of the measured crack lengths in every measurement was around 5%, and the accuracy of the measured energy of adhesion was evaluated to be around 90%. In this study, due to a special case that a flexible beam was mounted on a rigid substrate, equation (3) can further be simplified and the fracture toughness was approximately determined to be^52^

6 $${G}_{c}=\frac{3{E}_{2}{h}_{2}^{3}{L}^{2}}{8{a}^{4}{[1+0.64(\frac{{h}_{2}}{a})]}^{4}}$$

Equation (6) was used to calculated the interfacial fracture energy for all the Al/PI bonding experiments.

For comparison with fracture energy of homogeneous Al/Al and PI/PI interfaces, Al-Al and PI-PI direct bonding was conducted through Ar-FAB^20^ and water-vapor-assisted VUV treatments^18,19^, respectively. Detailed parameters of bonding experiments are listed in Table 3. After bonding, a double cantilever beam (DCB) test, where the thickness of two beams of the same material should be the same, was used to estimate fracture energy for homogeneous interface. Similar to the ADCB test, the DCB test was developed based on crack propagation in a cleaved bulk specimen of a linear elastic solid^57^, and the fracture energy can be calculated to be^44,57,58^

7 $${G}_{c}=\frac{3E{h}^{3}{L}^{2}}{16{a}^{4}}$$

where definitions of all the terms are the same as mentioned in equation (2). Equation (7) was used to estimate the interface fracture energy for all the Al-Al and PI-PI bonding specimens.

**Table 3:** Parameters of homogeneous and heterogeneous bonding for the ADCB test.

Bonding conditions	Al-Al	PI-PI	Al-PI
Surface treatment	Ar-FAB bombardment^20^ for 30 s	Water-assisted VUV irradiation process^18,19^ at 2.8RH% for 5 min	E-VUV treatment for the predetermined exposures (see Fig. S2c)
Contact pressure (MPa)	~2.07	~1.68	~4.00
Contact area (mm^2^)	400.0	400.0	100.0
Temperature (°C)	200	150	150
Bonding time (min)	10	10	10

### Reliability Test

A temperature humidity test was conducted at 85 °C and 85 RH% for 1000 hours, which met the requirement of industry standards (JESD22-A101 and MIL-STD-810F, Method 507.3). An environmental test chamber (IH-400, Yamato Ltd.) was used. After the storage time reached 1000 hours, the Al-PI specimens were retrieved from the chamber and interfacial strength was examined via the ADCB test. All the ADCB tests were done within 15 minutes to avoid unexpected influences like interfacial microstructural changes during cooling.

## Supplementary information

Supplementary Information

## Data Availability

All data generated or analyzed during this study are included in this published article (and its Supplementary Information files), or are available from the corresponding author on reasonable request.
