# Supplementary material for: Organic-Inorganic Solid-State Hybridization with High-Strength and Anti-Hydrolysis Interface
Source: Sci Rep. 2019 Jan 24;9:504. doi: 10.1038/s41598-018-37052-1 (PMC6345999; doi:10.1038/s41598-018-37052-1)
Supplement: Supplementary file 1 — Supplementary Information [file 41598_2018_37052_MOESM1_ESM.docx]

**Organic-Inorganic Solid-State Hybridization with High-Strength and Anti-Hydrolysis Interface**

Tilo H. Yang^1,2,^*, C. Robert Kao^1^ & Akitsu Shigetou^2^

^1^ Department of Materials Science and Engineering, National Taiwan University, Taipei 10617, Taiwan

^2^ National Institute for Materials Science (NIMS), Tsukuba, Ibaraki 305-0044, Japan

Corresponding Author

*Tilo H. Yang; E-mail: f03527057@ntu.edu.tw

**Supplementary Information**

**Estimation of Ethanol Exposure.** The ethanol exposure (*Γ*) is defined as:

$\Gamma=\rho\cdot t$ (1)

where the units of *Γ* are kg⋅s⋅m^-3^, *ρ* is the vapor density of ethanol (kg⋅m^-3^), and *t* is the VUV irradiation time (s). The percentage relative humidity (*RH%*) was recorded during E-VUV treatment; therefore, the ρ term can be obtained by:

$\rho=RH\%\cdot SVD$, (2)

where *SVD* is the saturation vapor density of ethanol (kg⋅m^-3^). If the temperature is sufficiently low (ca. <400 K) then viscous flow is expected to behave as molecular flow due to low kinetic energy of the gases and the negligible interaction forces among the gas molecules. The ideal gas law can be manipulated to estimate SVD from the saturation vapor pressure (*SVP*):

$PV=\left( \frac{m}{M} \right)RT$, (3)

where *P* represents the *SVP* of ethanol (Pa), *V* is the chamber volume (m^3^), *m* is the mass of ethanol molecules present (kg), *M* is the molar mass of ethanol (kg⋅mol^-1^), *R* is the universal gas constant (J⋅mol^-1^⋅K^-1^), and *T* is the temperature (K). Rearrangement of Eq. (3) and substitution of the *SVP* for the *P* term give:

$\frac{m}{V}=SVP\cdot\frac{M}{RT}=SVD$ (4)

Thus, the relation between *SVP* and *SVD* is identified. As the thermodynamics theory holds^1^, the *SVP* of any gases in a unary system can be approximated by the Clausius-Clapeyron equation by assuming the vapor phase behaves as an ideal gas and at a two-phase equilibrium. The nature of the two phases, vapor and liquid, is not specified in the Clausius-Clapeyron equation; therefore, this equation can generally be applied to all vapor-liquid phase boundaries. Eq. (5) is the form of the Clausius-Clapeyron equation employed most frequently in computing unary phase diagrams^1^.

$\ln\left( \frac{P_{2}}{P_{1}} \right)=\frac{{\Delta H}_{v}}{R}\left( \frac{1}{T_{1}}-\frac{1}{T_{2}} \right)$ (5)

Here, ΔH_v_ represents the enthalpy of vaporization (J/mol). Given that ethanol has a boiling point of 78.37°C, and the relationship for the ΔH_v_ of ethanol and temperature was adopted from the International Union of Pure and Applied Chemistry (IUPAC) chemistry database^2,3^ to the following.


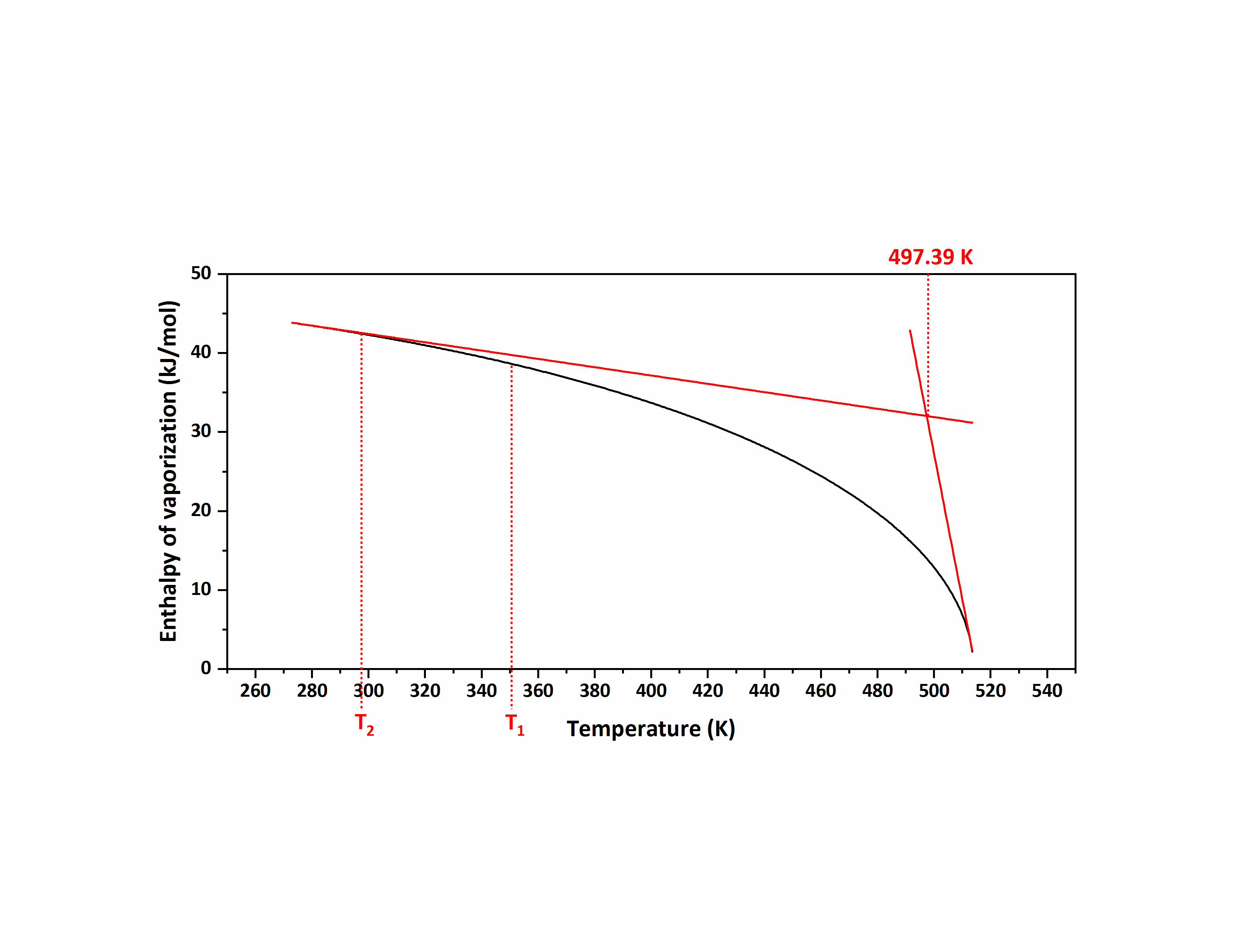


Relationship for the enthalpy of vaporization of ethanol and temperature.

In this diagram, two tangent lines are drawn, one from 273.00 K and the other from approximately the critical temperature of ethanol, which produces a point of intersection corresponding to 497.39 K. Both the boiling point (T_1_) and the processing temperature of E-VUV (T_2_) are located in the same interval and are much lower than 497.39 K; therefore, ΔH_v_ can be safely considered to be temperature-independent and averages at 42300 J/mol. Thus, the saturation vapor pressure of ethanol at 24°C can be calculated.

*T*_1_ = (78.37+273.15) K = 351.52 K; *P*_1_ = 101325 Pa

*T*_2_ = (24.00+273.15) K = 297.52 K; *P*_2_ = *SVP* at 24°C

Inserting these *T* and *P* values into the Clausius-Clapeyron Equation (5) produces:

$\ln\left( \frac{P_{2}}{101325 Pa} \right)=\frac{42300 J/{mol}}{8.31 J/{mol\cdot K}}\left( \frac{1}{351.52 K}-\frac{1}{297.52 K} \right)$ (6)

Thus,

$P_{2}=e^{-2.63}=7325.39 N\cdot m^{-2}=SVP at 24℃$ (7)

Setting this *SVP* value into Eq. (4) gives:

$SVD=7325.39 N/{m^{-2}}\cdot\frac{46.00\cdot{10}^{-3} {kg}/{mol}}{\left( 8.314J/{mol\cdot K} \right)\cdot\left( 297.52 K \right)}=0.14 kg\cdot m^{-3}$ (8)

Finally, using this *SVD* value in the combination of Eq. (1) and (2) yields:

$\Gamma=0.14RH\%\cdot t$ (9)

Eq. (9) offers an approximation for the exposure of ethanol at 24°C if the exposure time and relative humidity are known.


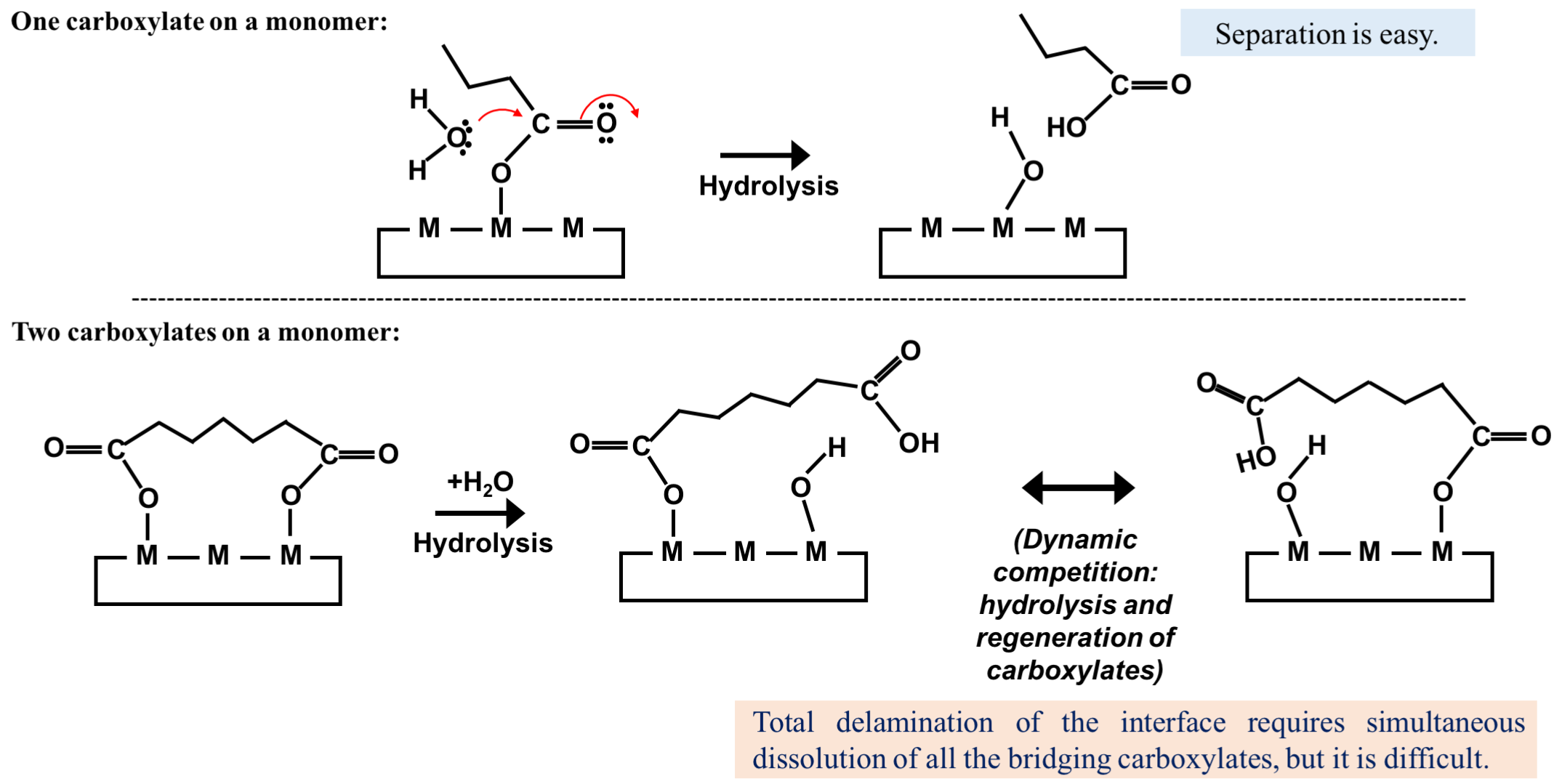


**Figure S1. Schematic of anti-hydrolysis interfacial architecture via dynamic competition of carboxylate.**

**
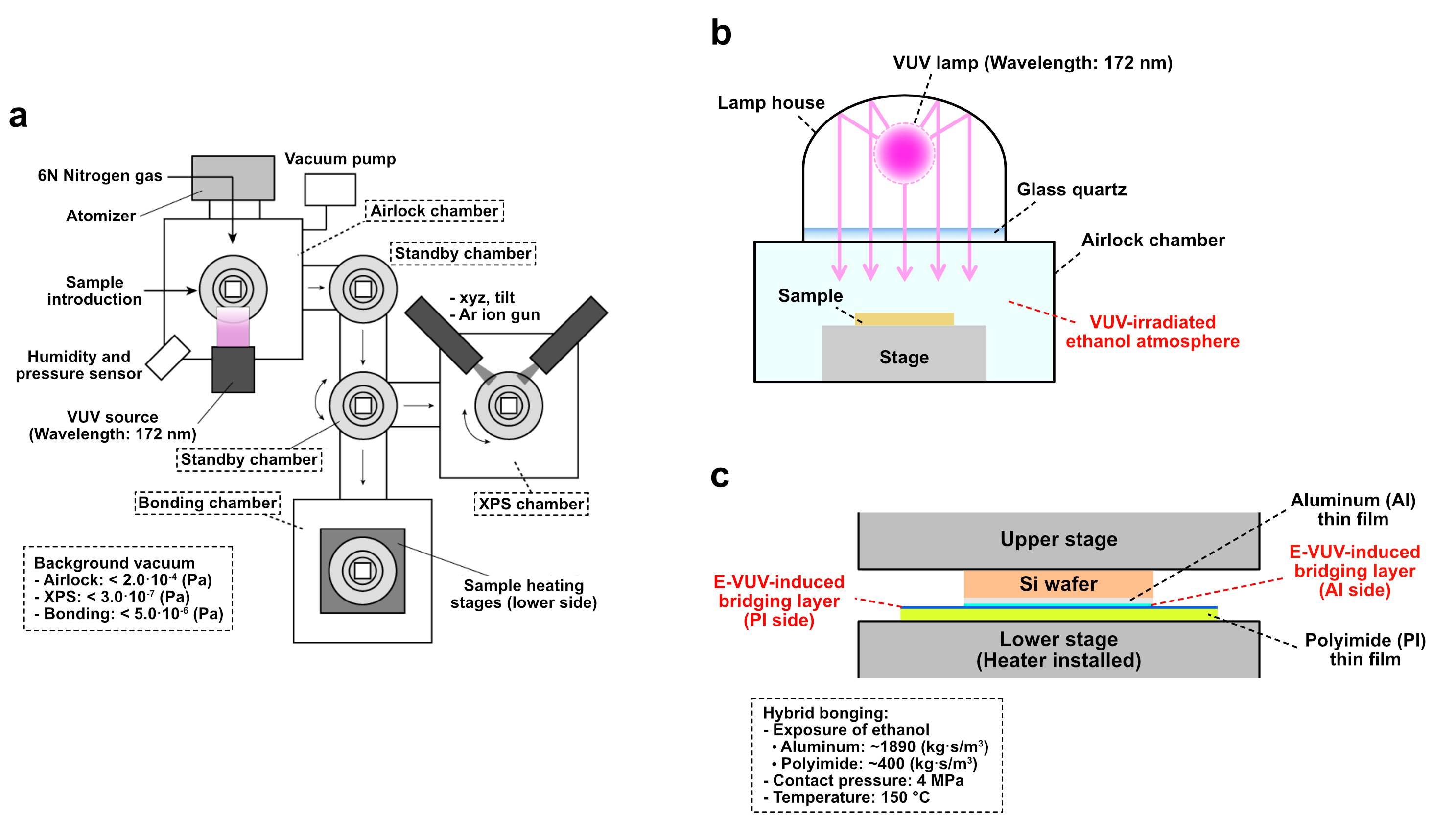
**

**Figure S2. Schematic of hybrid bonding/surface analysis apparatus and the E-VUV process.** (a) The apparatus comprises three chambers with different functions, including VUV irradiation (airlock chamber), XPS analysis, and hybrid bonding. The VUV chamber was installed with an ultrasonic atomizer that introduces an ethanol-containing nitrogen atmosphere into the VUV chamber. (b) and (c) show how the E-VUV treatment and hybrid bonding were conducted in the VUV and bonding chambers, respectively.

**
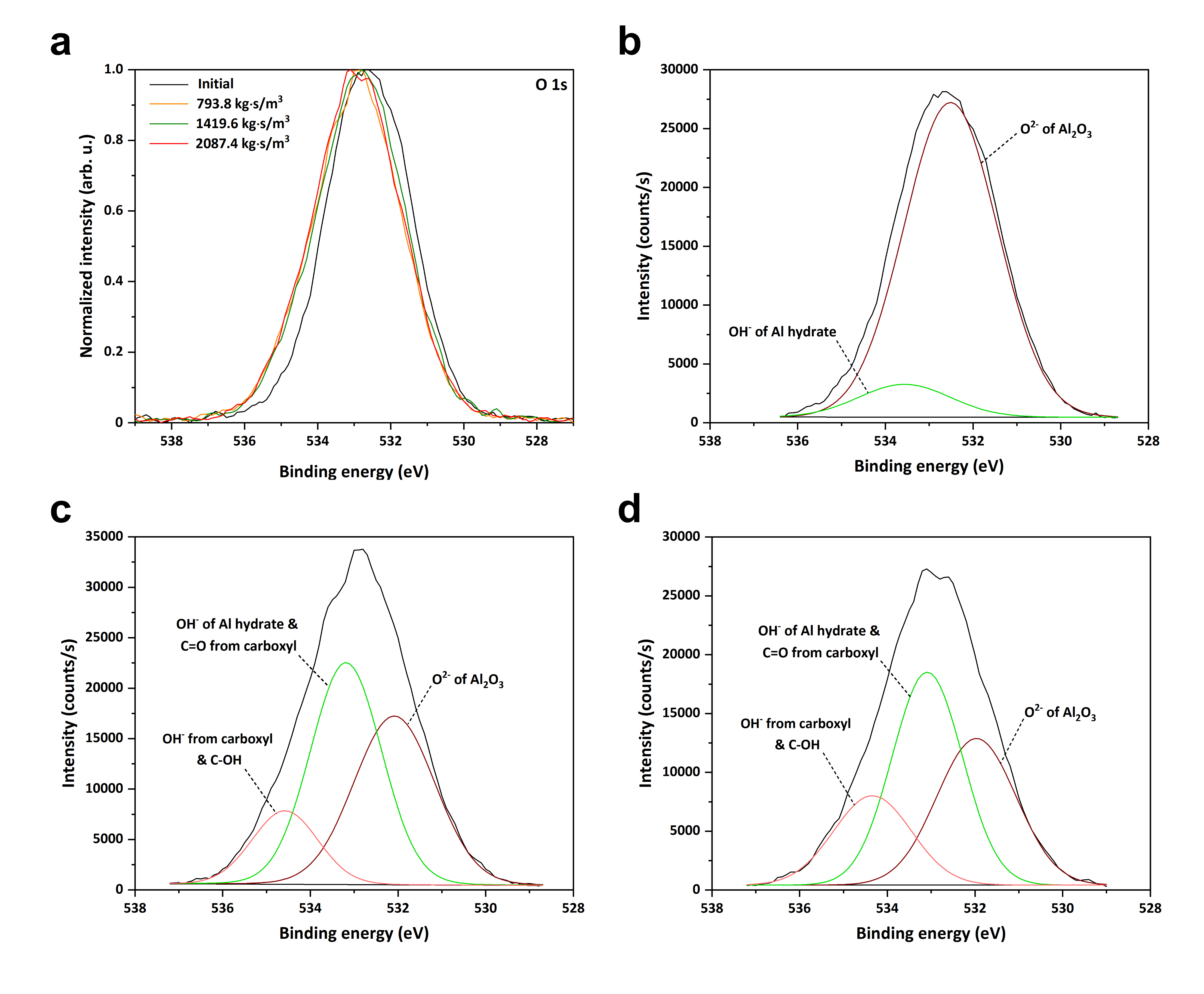
**

**Figure S3. Deconvolution analysis of XPS O 1s spectra for the E-VUV-treated aluminum.** The exposures shown here are the same as those shown in Fig. 2a. (a) XPS O 1s spectra with increase in the exposure. Spectra were normalized with respect to the highest intensity to emphasize the change in the chemical bonding status indicated by the difference in the binding energy of each peak, i.e. the shape of the synthesized wave. (b) to (d) show curve-fitting results for spectra with exposure of 0, 793.8, and 2087.4 (kg⋅s/m^3^) in (a).


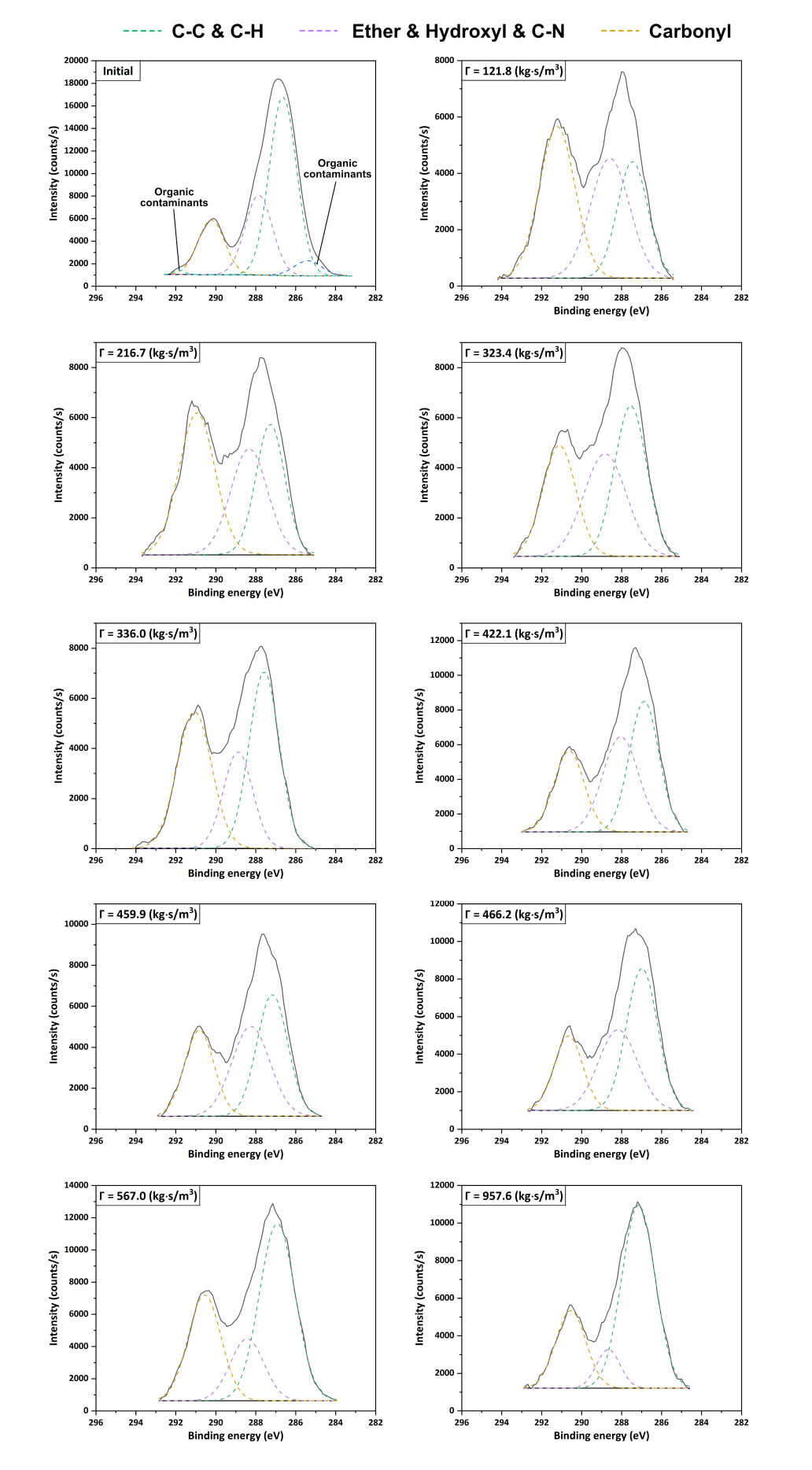


**Figure S4. Deconvolution analysis of XPS C 1s spectra for the E-VUV-treated PI.** The ratio of chemical components was estimated to obtain the results in Fig. 3a.


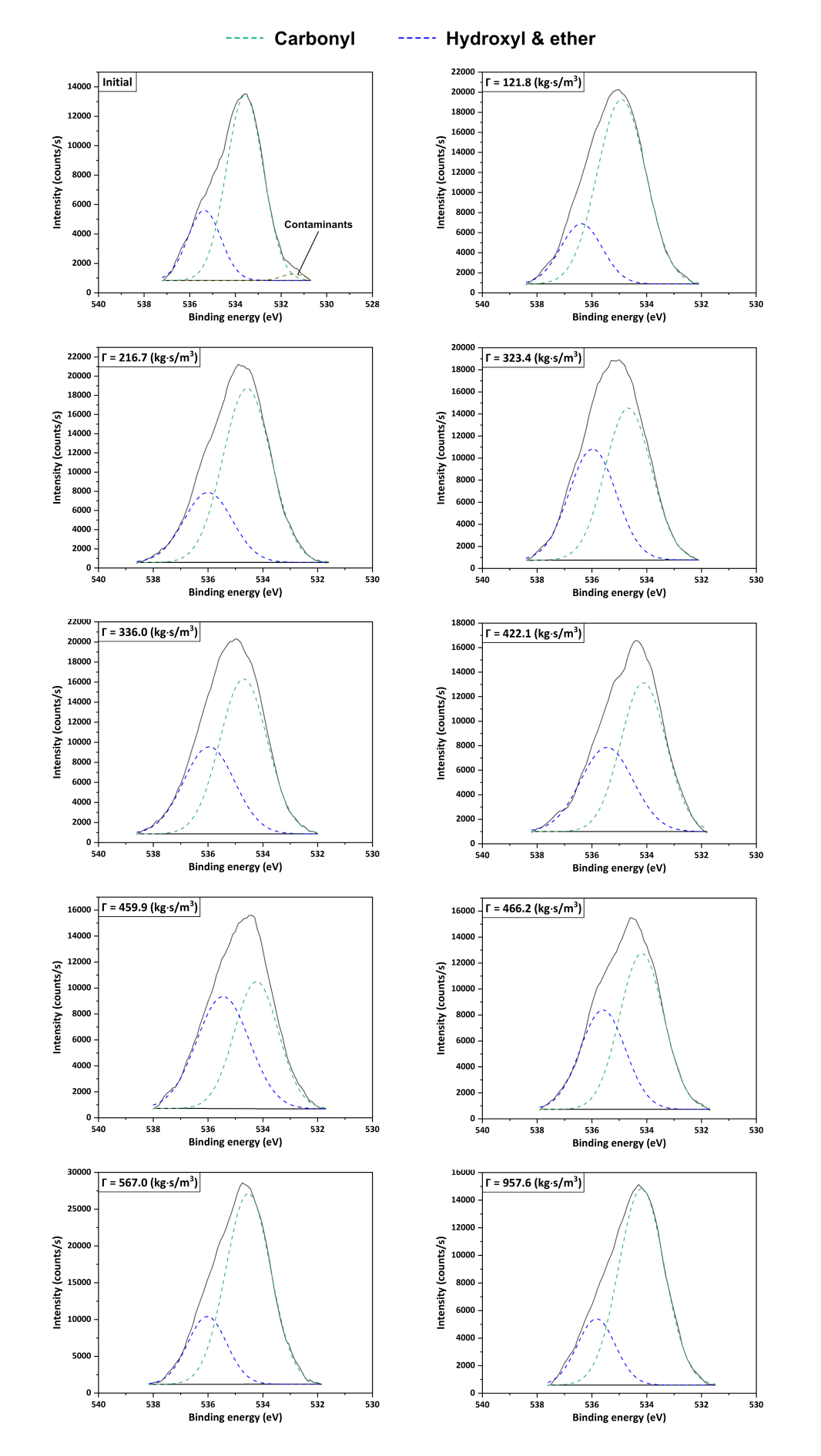


**Figure S5. Deconvolution analysis of XPS O 1s spectra for the E-VUV-treated PI.** The ratio of chemical components was estimated to obtain the results in Fig. 3b. The hydroxyl and ether groups at different exposures are estimated in Fig. S6.


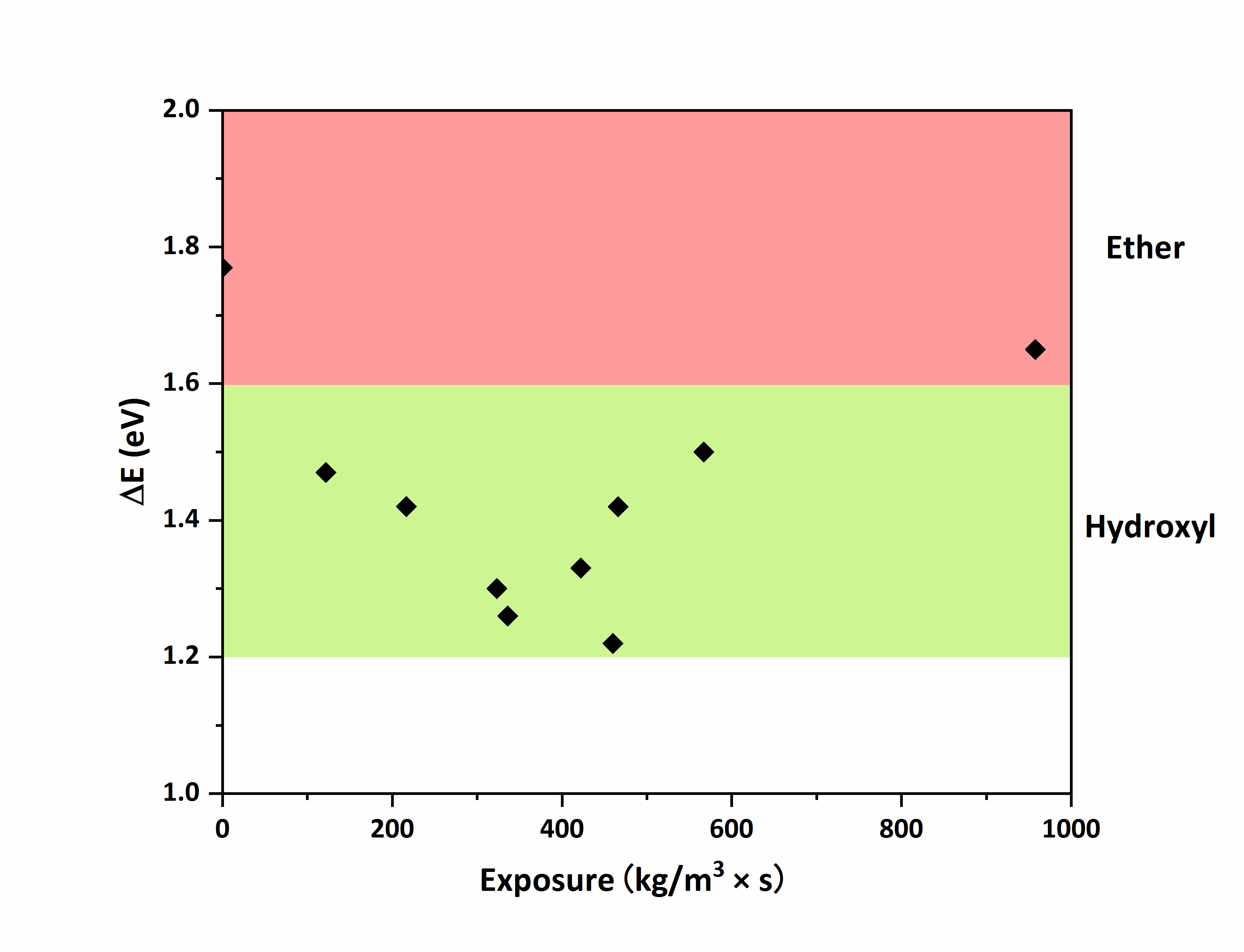


**Figure S6. Change of energy gap between the green and blue peaks in Fig. S5.** The peak positions of ether and hydroxyl in the XPS O 1s spectra were extremely close, which made identification difficult. In this diagram, the distribution of data points was divided into two regions that were marked with green and red background, respectively. Given that there were only carbonyl and ether groups at the initial stage^4^ and that neighboring atoms with low electronegativity result in nucleus of oxygen less exposed and decrease in binding energy^5-8^, this diagram can be used to determine which component was dominant among ether and hydroxyl groups; the data points located in the red region implied that ether groups were the dominant species on E-VUV-treated PI surface, whereas the green region implied hydroxyl-dominant.





**Figure S7. ATR-FTIR spectra for PI with increasing exposure.** The peaks at 2847 and 2913.4 (cm^-1^) arise from sp^3^ C-H stretching vibration of acyclic alkyl chains. These spectra again confirm the formation of acyclic alkyl with the E-VUV process, which is consistent with the conclusion obtained from Fig. 3a.

**
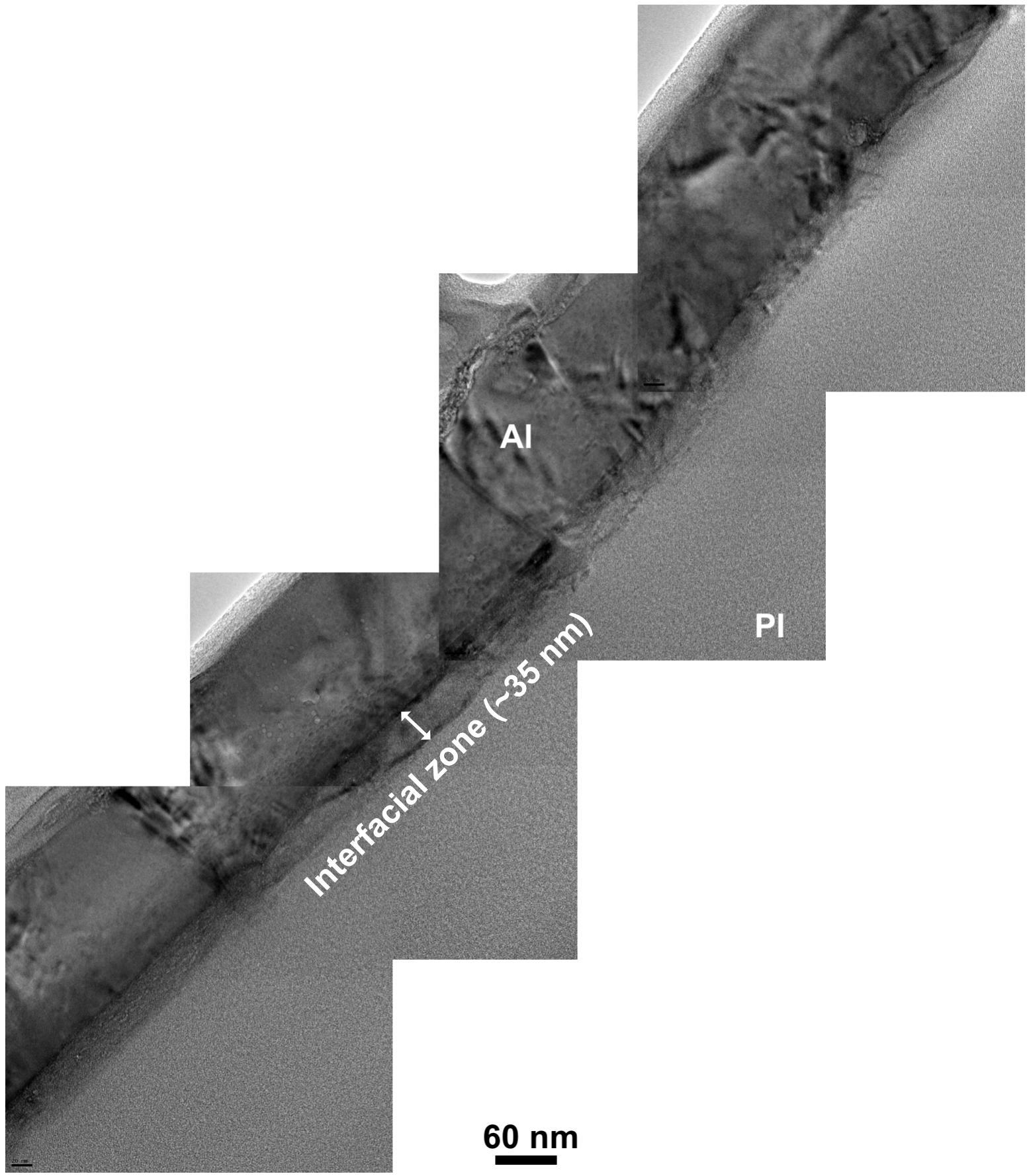
**

**Figure S8. Wide-range TEM image of the Al/PI interface after room temperature storage for 6 months.** The thickness of the interfacial zone was measured to be ~35 nm. No visible void was observed.

**References**

1. DeHoff, R. *Thermodynamics in Materials Science* (McGraw-Hill, New York, ed. 2, 1993), chap. 7.
2. Majer, V. & Kehiaian, H. V. *Enthalpies of vaporization of organic compounds: a critical review and data compilation*, V. Svoboda, Ed. (Blackwell, Oxford, 1985).
3. Chickos, J. S. & Acree Jr., W. E. Enthalpies of Vaporization of Organic and Organometallic Compounds, 1880-2002, *J. Phys. Chem. Ref. Data* **32**, 550 (2003).
4. Yang, H. W., Kao, C. R. & Shigetou, A. Fast atom beam- and vacuum-ultraviolet-activated sites for low-temperature hybrid integration. *Langmuir* **33**, 8413-8419 (2017).
5. Moulder, J. F., Stickle, W. F., Sobol, P. E. & Bomben, K. D. *Handbook of X-ray photoelectron spectroscopy* (Perkin-Elmer, MN, 1992).
6. López, G. P., Castner, D. G. & Ratner, B. D. XPS O 1s binding energies for polymers containing hydroxyl, ether, ketone and ester groups. *Surf. Interface Anal.* **17**, 267-272 (1991).
7. Fu, W., Shigetou, A., Shoji, S. & Mizuno, J. Low-temperature direct heterogeneous bonding of polyether ether ketone and platinum. *Mater. Sci. Eng. C* **79**, 860-865 (2017).
8. Shinohara, H., Mizuno, J. & Shoji, S. Studies on low-temperature direct bonding of VUV, VUV/O_3_ and O_2_ plasma pretreated cyclo-olefin polymer. *Sens. Actuators A* **165**, 124-131 (2011).
